# Genome-Wide Identification and Analysis of Class III Peroxidases in Allotetraploid Cotton (*Gossypium hirsutum* L.) and their Responses to PK Deficiency

**DOI:** 10.3390/genes10060473

**Published:** 2019-06-21

**Authors:** Pengfei Duan, Guo Wang, Maoni Chao, Zhiyong Zhang, Baohong Zhang

**Affiliations:** 1 Henan Collaborative Innovation Center of Modern Biological Breeding and Henan Key Laboratory for Molecular Ecology and Germplasm Innovation of Cotton and Wheat, Henan Institute of Science and Technology, Xinxiang 453003, Henan, China; dpf19830905@126.com (P.D.); 18336063077@163.com (G.W.); chaomaoni@126.com (M.C.); zhangb@ecu.edu (B.Z.); 2 Henan Collaborative Innovation Center of Water Security for Water Source Region of Mid-line of South-to-North Diversion Project and Henan Key Laboratory of Ecological Security for Water Source Region of Mid-line of South-to-North Diversion Project, Nanyang Normal University, Nanyang 473061, Henan, China; 3 Department of Biology, East Carolina University, Greenville, NC 27858, USA

**Keywords:** cotton, class III peroxidases, POD, gene duplication, nutrient deficiency

## Abstract

Class III peroxidases (PODs), commonly known as secretable class III plant peroxidases, are plant-specific enzymes that play critical roles in not only plant growth and development but also the responses to biotic and abiotic stress. In this study, we identified 198 nonredundant POD genes, designated GhPODs, with 180 PODs being predicted to secrete into apoplast. These POD genes were divided into 10 sub-groups based on their phylogenetic relationships. We performed systematic bioinformatic analysis of the POD genes, including analysis of gene structures, phylogenetic relationships, and gene expression profiles. The GhPODs are unevenly distributed on both upland cotton sub-genome A and D chromosomes. Additionally, these genes have undergone 15 segmental and 12 tandem duplication events, indicating that both segmental and tandem duplication contributed to the expansion of the POD gene family in upland cotton. Ka/Ks analysis suggested that most duplicated GhPODs experienced negative selection, with limited functional divergence during the duplication events. High-throughput RNA-seq data indicated that most highly expressed genes might play significant roles in root, stem, leaf, and fiber development. Under K or P deficiency conditions, PODs showed different expression patterns in cotton root and leaf. This study provides useful information for further functional analysis of the POD gene family in upland cotton.

## 1. Introduction

Peroxidases (EC 1.11.1.x) are encoded by multigenic families and are involved in several important physiological and developmental processes. Among them, class III peroxidases (EC 1.11.1.7), belonging to the haem peroxidase subfamily, exist only in plants and have an extremely widespread presence in the plant kingdom [1]. They are members of a large multigenic family with more than 200 members in switchgrass [2], 93 in *Populus* [3], 138 in rice [4], and 73 in *Arabidopsis* [5]. The nomenclature of Class III plant peroxidase is not unified and various abbreviations were reported, such as POX [6,7], GPX [8], Prx [5], ClassIIIPRX [2], and POD [9,10]. Hereafter, class III peroxidases were abbreviated as PODs.

PODs are involved in a broad range of physiological processes such as auxin metabolism, lignin and suberin formation, cross-linking of cell wall components, phytoalexin synthesis, defense against biotic or abiotic stress, cell elongation, and the metabolism of reactive nitrogen species and reactive oxygen species (ROS), throughout the plant life cycle from the early stage of germination to the final step of senescence [11,12,13]. It is probably due to their high number of enzymatic isoforms and to the versatility of their enzyme-catalyzed reactions.

PODs are mainly considered as secreted/apoplastic/cell wall proteins, but vacuolar isoforms also exist [14]. Apoplastic POD can be further classified into three major categories based on their chemical and physical association with cell walls and available extraction methods: water soluble and loosely ionically bound, tightly ionically bound, and covalently bound [15]. The specific function of each member of the family is still elusive. Furthermore, they usually show dual enzymatic activities. For example, some consume ROS and others produce ROS; some loosen the cell wall and others stiffen the cell wall [14]. Therefore, they play a pivotal role in cellular growth and response to biotic and abiotic stresses. The comprehensive researches are necessary to explore the role of POD in plant growth and defense.

Widely cultivated in more than 100 countries, cotton is considered one of the most important fiber-producing and economic crops around the world. Suboptimal phosphorus (P) and potassium (K) availability, widely present in agriculture, negatively influences cotton growth and development and reduces cotton fiber yield and quality [16,17]. PODs participate in cotton growth and development, cotton defense against biotic and abiotic stresses, and fiber development. For example, GhPOX1 play an important role during fiber cell elongation possibly mediated by ROS production [6]. The cotton flower-specifically expressed *pod*, predominantly in pollen, suggested that peroxidase is involved in the male reproductive processes of angiosperms [18]. Two PODs from cotton play a role in the oxidative burst response of cotton to bacterial blight [19]. Up to now, however, no genome-wide characterization of the *pod* family and their responses to PK deficiency has been performed in cotton. The recently published genome sequence of *Gossypium hirsutum* L. acc. TM-1, a tetraploid cotton species [20], provides us with a great opportunity to identify and characterize *pod*s in the cotton genome and to explore the expression profiles of *pod*s under PK deficiency conditions.

In the present study, we performed for the first time the comprehensive analysis and responses to PK deficiency of the *pod* family in *G. hirsutum*. A total of 198 non-redundant POD encoding genes were identified in the genome of *G. hirsutum*. and were subsequently subjected to a systematic genomic analysis, including studies on phylogenetic relationships, on chromosome location, on gene duplication status, on substitution rates, on gene structures, on expression profiling and secretion traits, and on responses to PK deficiency in cotton leaf and root. The differentiation of functions of GhPODs were predicted on the basis of the expression profiles of *pod* members and the phylogenetic analysis among the POD proteins in *G. hirsutum*. Additionally, we analyzed whether the expansion of the *pod* family in *G. hirsutum* was caused by segmental duplication and/or tandem duplication. In summary, our genome-wide analysis of the POD gene family will contribute to future studies on the functional differentiation of POD proteins in different physiological processes of *G. hirsutum*; the differential responses to PK deficiency will benefit the elucidation of the relationship of physiological processes such as root elongation and branching, leaf senescence, and ROS modulation and with specific POD isoenzymes under PK deficiency.

## 2. Materials and Methods

### 2.1. Sequence Retrieval for POD Proteins in Cotton

The local BLAST database was established with protein sequences of upland cotton (*G. hirsutum* L. acc. TM-1) whole genome (download from http://mascotton.njau.edu.cn). The protein sequences of POD family members in the genome of *Arabidopsis* were retrieved from the TAIR database (http://www.arabidopsis. org/). The candidate sequences of POD in cotton were acquired by BLASTP with each of the 73 different amino acid sequences of *Arabidopsis* POD gene family as query sequences (screening threshold value/E-Value: 1e^−10^). To verify the reliability of the initial results, the acquired candidate sequences were further submitted against PFAM (http://pfam.xfam.org/) to verify the domains for identifying the POD gene family members in cotton. The theoretical molecular weights (MWs) and isoelectric points (pIs) of the proteins were collected through an online program (http://www.ebi.ac.uk/Tools/seqstats/emboss_pepstats/).

### 2.2. Phylogenetic Analysis

Multiple sequence alignments were conducted on the amino acid sequences of POD proteins in *G. hirsutum* genomes using Cluster W of MEGA 5.0 software with the default settings [21]. Subsequently, the software was employed to construct an unrooted phylogenetic tree based on alignments using the Neighbor-Joining (NJ) method with the following parameters: model (p-distance), bootstrap (1000 replicates), and gap/missing data (pairwise deletion).

### 2.3. Gene Structure Analysis

The genomic and CDS sequences of cotton PODs, extracted from *G. hirsutum* genome databases, were compared by using the Gene Structure Display Server program (http://gsds.cbi.pku.edu.cn/) to infer the exon/intron organization of POD genes.

### 2.4. Analysis of Chromosomal Location and Gene Duplication

Information about the physical locations of all POD genes on chromosomes was obtained through BLASTn searches against the *G. hirsutum* genome database. All GhPOD genes were then mapped on the chromosomes using the software MapInspect (http://mapinspect.software.informer.com). The detection of POD gene duplication events was also carried out and paralogous POD gene pairs were identified based on the alignment results. The criteria were as follows: the shorter sequence covers over 80% of the longer sequence after alignment and the minimum identity of aligned regions is equal to or above 80%. In addition, to explore the selection pressures among POD duplicated genes, we calculated the nonsynonymous mutation rate (Ka), synonymous mutation rate (Ks), and Ka/Ks values for the duplicated gene pairs with Mega 5.0.

### 2.5. Cotton Culture and Expression Analysis of POD Genes under PK Deficiency

Cotton (*G. hirsutum* L. TM-1) was planted in a growth chamber (day/night of 14/10 h with temperature 30/25 °C and photo intensity 450 µmol/m2·s) under liquid culture. The solution composition was as follows (mmol/L): 2.5 Ca(NO_3_)_2_, 1 MgSO_4_, 0.5 NH_4_H_2_PO_4_, 2.5 KCl, 2 NaCl, 2 × 10^−4^ CuSO_4_, 1 × 10^−3^ ZnSO_4_, 0.1 EDTA-FeNa, 2 × 10^−2^ H_3_BO_3_, 5 × 10^−6^ (NH_4_)_6_Mo_7_O_24_ and 1 × 10^−3^ MnSO_4_. The seedlings with four expanded leaves were treated separately with the original solution (control), low K solution (0.05 KCl with NaCl to balance the Cl ion and others the same as in the original solution), and low P solution (0.005 NH_4_H_2_PO_4_ with NH_4_Cl to balance the NH_4_^+^ and others the same as in the original solution). On the 7th day of treatment, the third leaf from the uppermost was counted and the young roots of all treatments were sampled and stored in −80 °C for RNA extraction and gene expression analysis.

Expression profiles of POD genes response to PK deficiency were analyzed by using the Illumina Hiseq2000 (Illumina, San Diego, CA, USA) to perform high-throughput RNA-seq of the root and leaf of control, P deficiency, and K-deficiency. In total, 26.95 Gb of raw RNA-seq data were generated (BGI-Tech., Shenzhen, China). RNA-seq reads were mapped to the cotton genotype TM-1 genome using Tophat (v2.0.8; Top Hat, Toronto, Canada). To measure the gene expression level in sampled tissues, we calculated the expression of each gene using FPKM (Fragments per Kilobase of exon model per Million mapped reads) with Cufflinks (v2.1.1; http://cole-trapnell-lab.github.io/cufflinks/). We analyzed the POD gene expression changes in root and leaf under control, P-deficiency, and K-deficiency by using software MultiExperiment Viewer (MeV; http://mev.tm4.org).

### 2.6. Localization of POD Proteins

Secretion of POD proteins to the apoplast or to the vacuole were predicted by combinations of using SignalP (www.cbs.dtu.dk/services/SignalP/) with a signal peptide, SecretomeP (www.cbs.dtu.dk/services/SecretomeP) without a signal peptide and TargetP (www.cbs.dtu.dk/services/TargetP). The secreted POD proteins were further investigated in the xylem saps, separately, from field cotton [8] and chamber cotton.

## 3. Results

### 3.1. Identification of POD Genes

We used the 73 *Arabidopsis* POD genes to acquire 264 cotton POD genes by BLASTP and further verified their domains with PFAM. A total of 198 non-redundant POD genes with conserved POD domains were identified in cotton. This number is greater than that in *Arabidopsis* (73) [5], *Populus* (93) [3], Chinese Pear (94) [22], maize (119) [23], and rice (138) ([4]; but it was similar with that in switchgrass (200) [2]. For convenience, we assigned names to these POD genes (GhPOD01-198) according to their chromosomal positions. The length of the 198 newly identified POD proteins varies from 160 to 1098 amino acid (aa) with an average of 332 aa. There is only one POD containing more than 672 aa. The isoelectric point (PI) varied from 4.12 to 10.50 with a mean of 7.73 and >7.0 of 67.2% POD proteins. Other information of chromosomal location, molecular weight (MW) gene size, coding sequence (CDs) size of each GhPOD gene/protein is shown in Table 1.

### 3.2. Phylogenetic Analysis

**Figure 1:**
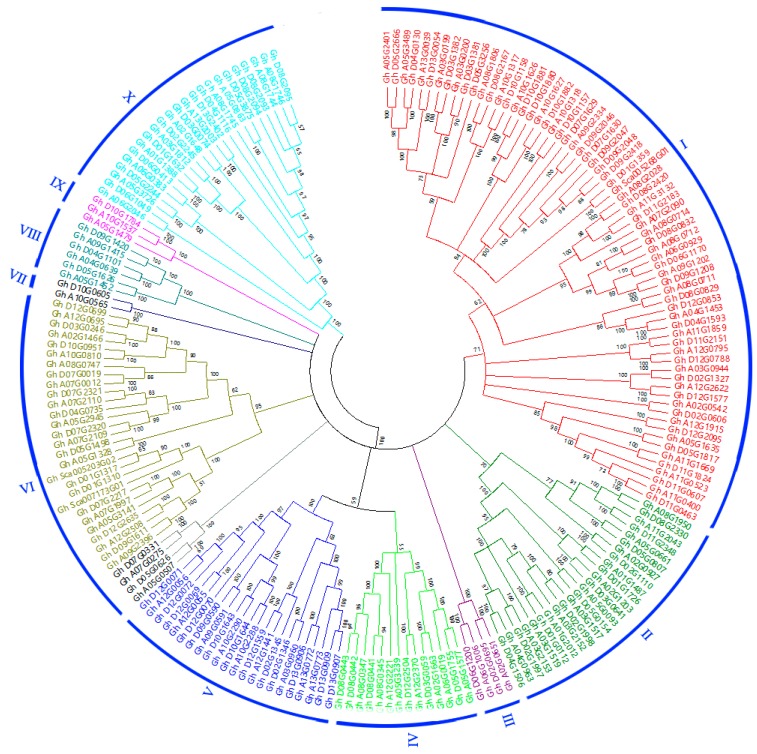
Phylogenetic relationship of the 198 identified upland cotton POD genes. Unrooted tree constructed using MEGA5.02 by the Neighbor-Joining (NJ) method. Bootstrap values (above 50%) from 1000 replicates are indicated at each node. The tree shows 10 major phylogenetic subfamilies (subfamilies I to X).

**Figure 2:**
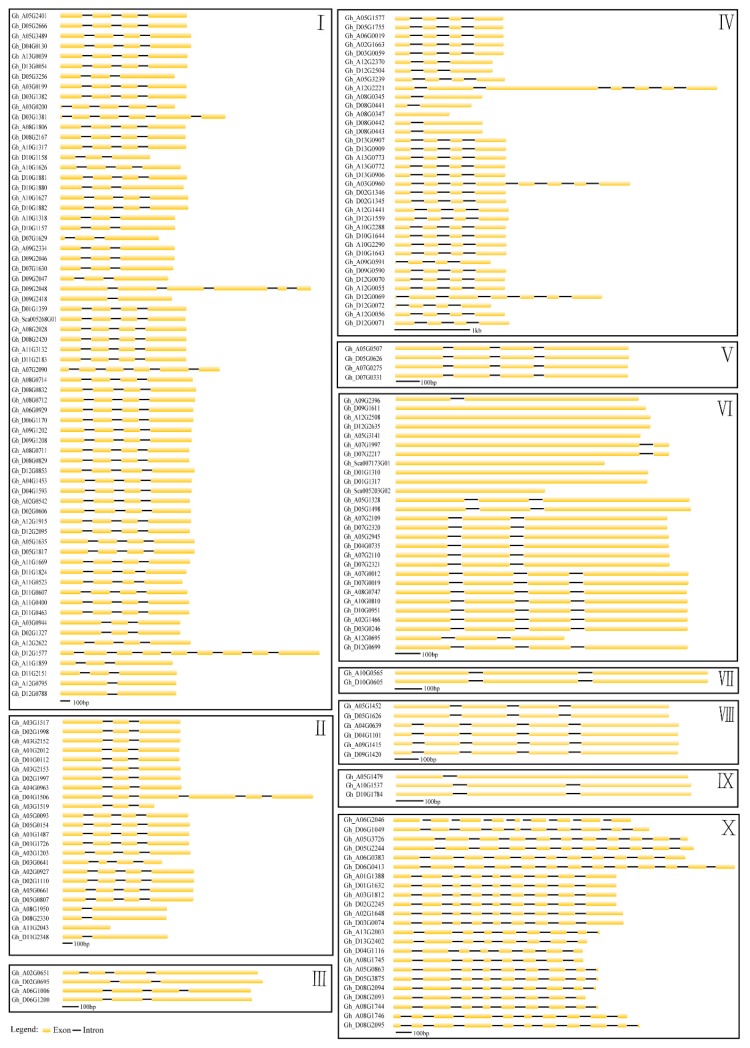
Exon–intron structures of the 198 identified upland cotton POD genes. Exons and introns are indicated by yellow cylinder bars and black lines, respectively.

The phylogenetic tree was constructed with the NJ method based on multiple sequence alignment of entire amino acid sequence of 198 upland cotton POD protein sequences in order to acquire a better understanding of evolutionary history and the phylogenetic relationship of POD in upland cotton. Based on the phylogenetic tree (Figure 1), we identified 10 major clusters with high bootstrap probabilities (BPs) ranging from 59 to 100%, among them six clusters had 100% BPs, one had 95% BPs, and two had 71% or nearly 71% BPs (Figure 1). The POD genes were not evenly distributed in some groups in upland cotton, Cluster I had the most members (68), which could be divided into seven subgroups, but Cluster VII only had two members (Figure 1 and Figure 2).

### 3.3. Gene Structures

POD genes with classical conserved intron/exon gene structure were observed. The coding sequence of 100 of the 198 peroxidase genes are disrupted by three introns at conserved positions (Figure 2). However, variations in this basic gene structure were observed for another 98 of the family, implicating loss of one or more introns (64) or gain of one or more introns (34). Forty genes lost one of the three putative ancestral introns, while fifteen genes lost two introns. Additionally, nine genes (among them, eight genes were closely related, belonged to VI subgroup) were devoid of any introns. In comparison with the classical three introns, the number of POD genes gaining one to nine more introns (except eight), were 5, 3, 2, 4, 13, 2, 4, and 1, respectively. These differences may be derived from a single intron loss or gain events during the long evolutionary period. In addition, 23 of 24 genes with more than seven introns constitute group X, which contains the largest numbers of introns. Sub-clusters with conserved intron/exon gene structure were also observed.

### 3.4. Chromosomal Location and Gene Duplication

To investigate the genome organization and distribution of GhPODs on different subgenomes and chromosomes (CH) in upland cotton, a chromosome map was constructed. Among 198 GhPOD genes, 94 and 101 were located in subgenomes A and D, respectively. The other three POD genes were located on scaffolds. GhPOD genes unequally distributes in both subgenome A and D. For subgenome A, CHA5 has the most POD members (15), and ChA1 and CHA4 have the least but same members (3); for subgenome D, CHD12 has the most POD members (12), and CHD6 has the least members (4). In addition, the majority of chromosomes, especially for subgenome A, exhibit a relatively high density of GhPOD genes, which tends to assemble at the bottoms or the tops, such as CHA3, 5, 7, 8, 12 and CHD3, 5, 8, 9.

**Figure 3:**
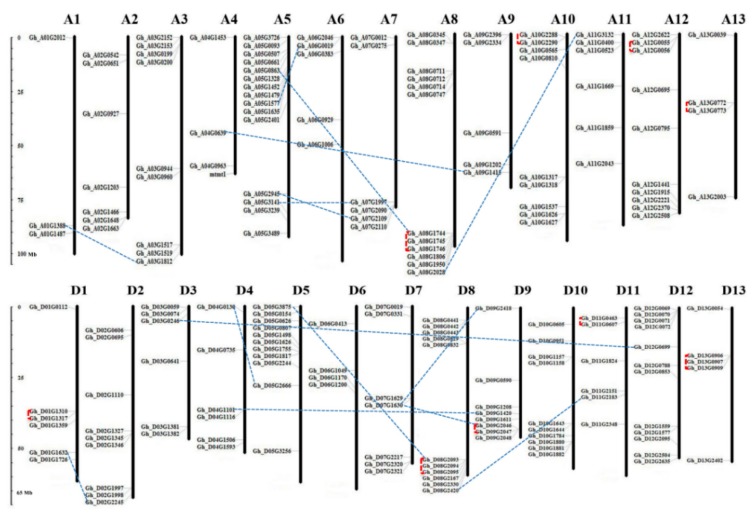
Chromosomal location and gene duplication events of 198 POD genes on 26 upland cotton chromosomes. Chromosome types and numbers are indicated at the top of each bar. The scale on the left is in mega-bases. The gene ID on the left side of each chromosome correspond to the approximate locations of each POD gene. The segmentally duplicated genes are connected by dashed blue lines, and the tandemly duplicated gene clusters are marked by red square bracket with dashed line.

Gene duplications, including segmental and tandem duplication, are one of the primary driving forces in genome evolution [24]. In this study, 27 duplicated gene pairs were identified (Figure 3, Table 2); among them, 12 and 15 GhPOD gene pairs were in subgenome A and subgenome D, respectively. A total of 15 gene pairs (29 genes) were localized to segmentally duplicated regions, while 9 gene clusters (12 gene pairs or duplication events; 21 genes) are arranged in tandem repeats [Segmental duplication (Gh_D07G1630/Gh_D09G2418) (I); (Gh_D07G1630/Gh_D09G2046) (I); Gh_A08G2028/Gh_A11G3132) (I); Gh_D08G2420/Gh_D11G2183 (I); Gh_D04G0130/Gh_D05G2666 (I); Gh_A05G1577/Gh_A06G0019 (IV); Gh_D03G0246/Gh_D12G0699 (VI); Gh_D04G1101/Gh_D09G1420 (VI); Gh_A04G0639/Gh_A09G1415 (VI); Gh_A05G2945/Gh_A07G2109 (VIII); Gh_A05G3141/Gh_A07G1997 (VIII); Gh_D01G1632/Gh_D02G2245 (X); Gh_D05G3875/Gh_D08G2093 (X); Gh_A01G1388/Gh_A03G1812 (X); Gh_A05G0863/Gh_A08G1744 (X); Tandem duplication (Gh_D09G2046/Gh_D09G2047 (I); Gh_D11G0463/Gh_D11G0607 (I); Gh_D13G0906/Gh_D13G0907/Gh_D13G0909 (IV); Gh_A10G2288/Gh_A10G2290 (IV); Gh_A12G0055/Gh_A12G0056 (IV); Gh_A13G0772/Gh_A13G0773 (IV); Gh_D01G1310/Gh_D01G1317 (VI); Gh_A08G1744/Gh_A08G1745/Gh_A08G1746 (X); Gh_D08G2093/Gh_D08G2094/Gh_D082095 (X)].

To explore the selection pressures among duplicated POD genes, we calculated the Ka, Ks, and Ka/Ks values for 27 identified gene pairs (Table 2). In general, Ka/Ks > 1 indicates positive selection, Ka/Ks = 1 indicates neutral selection, and Ka/Ks < 1 indicates negative selection. The Ka/Ks ratios of most GhPOD gene pairs were <1 except for two pairs (GhPOD55 and GhPOD56; GhPOD56 and GhPOD57) with Ka/Ks = 1, suggesting that these gene pairs were evolved under negative selection in upland cotton.

### 3.5. Responses of POD Genes to PK Deficiency in Cotton Roots and Leaves

Among 198 GhPOD genes, the expression of 30 genes was not detected in all samples including roots and leaves under control conditions, and under K and P deficient conditions. In roots, K and P deficiency, respectively, induced 10 and 11 POD gene expression from zero to slight level (FPKM value < 1). In leaves, K and P deficiency, respectively, induced 28 and 11 POD gene expression from zero to slight level (FPKM value < 1 for most of them). For roots, compared with controls, 12 and 42 POD gene expression, respectively, was above 2-fold and below 0.5-fold under K deficiency; the expression of 18 POD genes, respectively, was above 2-fold and below 0.5-fold under P deficiency. In leaves, compared with controls, the expression of 25 and 16 POD genes, respectively, was above 2-fold and below 0.5-fold being subjected to K deficiency; the expression of 12 and 33 POD genes, respectively, was above 2-fold and below 0.5-fold being subjected to P deficiency. The same POD gene expressed itself with obviously different patterns in leaves or roots under K or P deficiency. For example, under K deficiency, the expression of Gh_A08G1806 was 0.2 times as much as the controls; however, under P deficiency, it was 6.1 times as much as controls in roots. K deficiency induced the expression of Gh_A08G1806 from zero to 1.3 FPKM, but P deficiency did not change its transcription level.

### 3.6. Secret Traits of Cotton POD

**Figure 4:**
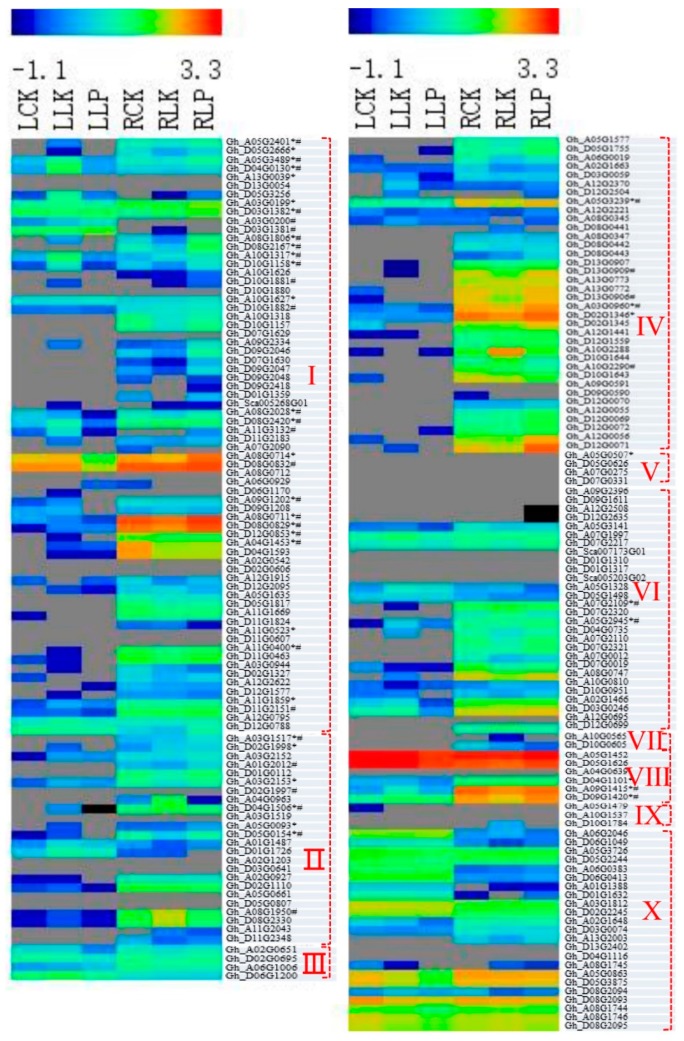
Responses of gene expression in cotton root and leaf to PK deficiency. Values are log_10_^FPKM^. * the POD protein was detected in the field cotton xylem sap; ^#^ the POD protein was detected in the greenhouse cotton.

Among 198 GhPOD genes, 142 POD enzymes were predicted with signal peptide by using SignalP, 147 with signal peptide by using TargetP, another 38 POD enzymes were predicted being secreted into apoplast with SecretomeP among no signal peptides by SignalP prediction (Table 1). In xylem sap, 61 POD enzyme isoforms were identified. Among them, 31 isoforms were found not only in field conditions but also in greenhouse conditions (Figure 4).

## 4. Discussion

### 4.1. Identification of Cotton POD Genes and Their Expansion

Members of POD gene family are involved in the regulation of a variety of biological processes. POD proteins are classified into apoplast type and vacuole type [25]. Apoplast type PODs participate in plant cell wall lignification, defense to abiotic and biotic stresses, plant growth and development, etc. The majority of PODs (90%) was predicted to be secreted to apoplast by using SignalP plus SecretomeP, and 61 PODs in total were detected in cotton xylem sap from adult cotton plants in the field and cotton seedlings in the greenhouse, indicating their different roles in cotton growth and development. The predictive tools for localization show very different results, indicating the fact that plant localization signals are very variable.

Previously, Delannoy et al. (2003) characterized nine POD genes, found them showing differential expressions in response to the pathogen and suggested that they may have various functions in cotton defense to bacterial blight disease [9]. Furthermore, Delannoy et al. (2006) analyzed 12 POD genes from cotton and found two of them played a role in the oxidative burst response of cotton to bacterial blight [19]. Mei et al. (2009) also investigated 10 POD genes in cotton fiber development [6].

Systematic and comprehensive analyses of POD gene families have been published for *Populus trichocarpa* [3], *Zea mays* [23], *Arabidopsis thaliana* [5], and *Oryza sativa* [4]. The genome data of allotetraploid upland cotton [20] provides a useful tool for analysis of the upland cotton POD gene family. In our study, 198 POD genes were identified and characterized in upland cotton. The number in the upland cotton is higher than that in *Arabidopsis* (73) [5], Poplar (93) [3], Chinese Pear (94) [22], maize (119) [23], and rice (138) [4] and similar to that in switchgrass (more than 200) [2]. This is probably due to the fact that upland cotton and switchgrass are tetraploid with larger genomes containing two-type sub-genomes (respectively, 26 chromosomes, A and D; 18, A and B), and that *Arabidopsis*, maize and rice were diploid with smaller genomes (respectively, 5 chromosomes; 10; 12). However, this cannot explain the fact that the tetraploid *Populus* with 19 chromosomes in the genome has only 93 POD isoforms.

Gene duplications are one of the primary driving forces in the evolution of genomes and genetic systems [24]. Certain studies have shown that segmental duplication was largely responsible for the expansion of cotton gene families such as the TCP transcription factors in *G. raimondii*, YABBY and GhHsp20 in *G. hirsutum* [26,27,28]. By contrast, tandem duplication has contributed significantly to the expansion of this gene family in poplar [3]. However, for nsLTPs, both tandem and segmental duplication contributed to its expansion in *G. arboreum* and *G. hirsutum*, while tandem duplication was the dominant pattern in *G. raimondii* [29]. Interestingly, in this study, we determined that the number of GhPOD genes involved in segmental duplication and tandem duplication is similar, suggesting that both segmental and tandem duplications were equal contributors to the expansion of the POD gene family in upland cotton. It showed a similar gene duplication with POD genes in maize [23]. The Ka/Ks < 1 of the most GhPOD duplicated pairs showed that negative selection may be largely responsible for maintaining the functions of upland cotton POD enzymes.

Phylogenetic analysis of the GHPOD gene family revealed that the exon/intron structures of these genes are relatively conserved due to one half of the GhPOD genes with the 4 exons/3 introns structures. Similarly, 48 of the 73 (65.8%) peroxidase encoding genes in *Arabidopsis* consist of 4 exons/3 introns [5], and 38 of 138 (27.5%) in rice constitute this structure. It suggests a common ancestral gene with a classical pattern of 4 exons/3 introns [4]. Many studies have shown that introns were specifically inserted into plants and were retained in the genome during the course of evolution [30]. Another half of GhPOD genes gained or lost one or more introns from the POD coding region in a subfamily specific manner, and what is most serious is that some genes contain no introns. This extreme case for POD genes also exist in *Arabidopsis* [5], rice [4], maize [23], which might be explained either by the loss of all introns or by the occurrence of a reverse transcription event followed by the integration of the cDNA copy back in the genome, as described in mammals, yeast, and maize [31,32]. It is well known that the structural diversity of genes drives the evolution of multigene families. Also, the differences in these characteristics detected between different subfamilies suggest that upland cotton POD members are functionally diversified.

### 4.2. Expression Profiles of GhPOD Genes

**Figure 5:**
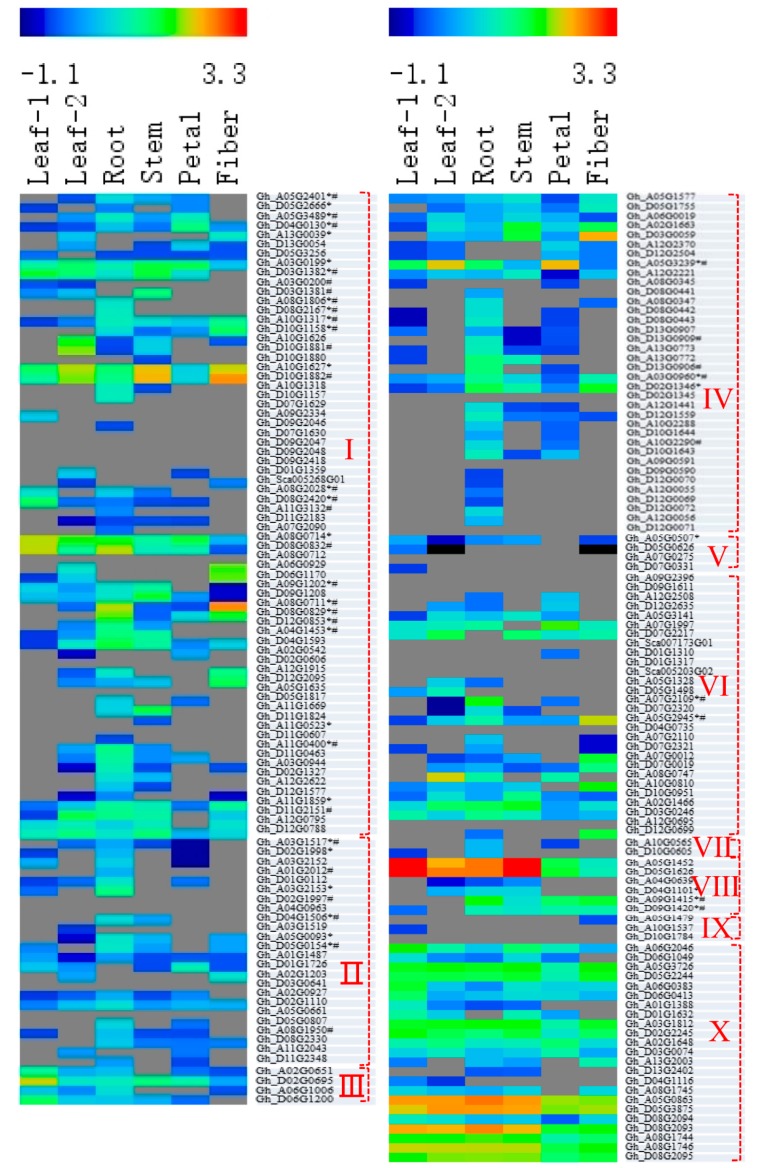
Expression profiles of POD genes across different cotton tissues. Values are log_10_^FPKM^. * the POD protein was detected in the field cotton xylem sap; ^#^ the POD protein was detected in the greenhouse cotton. Sources of the samples are as follows: leaf-1 (true leaves, Accession: SRX849561); leaf-2 (leaves at 2-week-old plants, Accession: SRX797901); root (roots at 2-week-old plants, Accession: SRX797899); stem (stems at 2-week-old plants, Accession: SRX797900); petals (petals of mature flowers, Accession: SRX797903); fibers (fibers of 25 days post-anthesis).

Gene expression patterns can provide important clues about gene function. We used publicly available [20] (https://www.ncbi.nlm.nih.gov/sra/?term=PRJNA248163) and our own genome-wide transcripts profiling data from upland cotton tissues as a resource to investigate the expression patterns of GhPODs. Based on the public data, 28 of the 198 identified GhPOD genes were not expressed in leaves, roots, stems, petals or fibers (Figure 4 and Figure 5), indicating their functional loss among all organs. In comparison between our results and the results of public data by using data from normal culture conditions, 56, 35, and 19 of the 198 GhPOD genes, respectively, exhibited no expression in leaves or/and roots (Figure 4 and Figure 5). It indicated that a part of GhPODs are expressed coincidently under different conditions or at different developmental stages. Few POD genes demonstrate tissue or organ specificity. In the *Arabidopsis* genome, 73 POD genes have been annotated, 65 of which were expressed in various tissues, and only three (AtPrx12, AtPrx62, AtPrx65) identified as specific to roots [33]. In the upland cotton genome, 17 of 198 POD genes were identified as specific to roots. However, only 4 and 1 were expressed in leaves and fibers, respectively (Figure 4 and Figure 5).

PODs are expressed in different patterns when facing different biotic and abiotic stresses [19,23,34,35]. This was also confirmed in roots and leaves when plants were subjected to K or P deficiency. For example, expression level of gene Gh_D10G1158 decreased obviously in roots under K deficiency and was about 30% of the controls; under P deficiency, its expression was increased obviously and was 202% as much as controls. Conversely, its expression level was increased by 20.9-fold in the leaf under K deficiency, but very few of them show expression changes under P deficiency. Additionally, different subfamily genes showed obviously different responses to K or P deficiency. In comparison with the controls, 16.7% and 45.2% of GhPOD genes had, respectively, more than 2 and less than 0.5 times the expression level in the root under K deficit in the I subfamily; 48.0% and 33.3% of GhPOD genes had, respectively, more than 2 and less than 0.5 times the expression level in the root under P deficit in the I subfamily. Interestingly, few GhPOD genes with higher expression level with FPKM > 30 showed changes of more than 2 or less than 0.5 times in leaf or root under K or P deficit, indicating that these genes play important roles in the maintenance of basic plant growth.

## Figures and Tables

**Table 1:** The 198 POD genes identified in cotton and their sequence characteristics and location.

Protein Name/ID	Chr Location	Gene/CDS Size (bp)	PL (aa)/MW (Kda)/PI	SignalP/SeretomeP/TargetP
GhPOD01/Gh_A01G1388	chrA01:87059677-87066826	7150/867	288/31.86/5.64	N/S/-
GhPOD02/Gh_A01G1487	chrA01:90340312-90341927	1616/972	323/34.9/5.69	S/N/S
GhPOD03/Gh_A01G2012	chrA01:175229-177095	1867/975	324/35.23/9.57	S/N/-
GhPOD04/Gh_A02G0542	chrA02:8131024-8132267	1244/1002	333/36.13/4.12	S/N/S
GhPOD05/Gh_A02G0651	chrA02:10404884-10407370	2487/1029	342/37.15/5.16	S/N/S
GhPOD06/Gh_A02G0927	chrA02:35288344-35290345	2002/1020	339/37.96/8.3	S/N/S
GhPOD07/Gh_A02G1203	chrA02:69325920-69327130	1211/984	327/35.33/8.2	N/N/S
GhPOD08/Gh_A02G1466	chrA02:80770130-80772880	2751/993	330/36.02/9.44	S/N/S
GhPOD09/Gh_A02G1648	chrA02:82884707-82887518	2812/909	302/33.98/9.11	N/S/-
GhPOD10/Gh_A02G1663	chrA02:82988074-82989301	1228/954	317/34.25/9.27	S/N/S
GhPOD11/Gh_A03G0199	chrA03:3093785-3095982	2198/969	322/34.57/9.93	S/N/S
GhPOD12/Gh_A03G0200	chrA03:3102427-3103937	1511/744	247/26.58/5.73	N/S/C
GhPOD13/Gh_A03G0944	chrA03:60522384-60523564	1181/1008	335/36.76/5.19	S/N/S
GhPOD14/Gh_A03G0960	chrA03:61923097-61977652	54556/1971	656/71.66/8.68	S/N/S
GhPOD15/Gh_A03G1517	chrA03:95777005-95778170	1166/984	327/35.68/9.58	S/N/S
GhPOD16/Gh_A03G1519	chrA03:95814422-95816062	1641/723	240/25.77/6.99	S/N/S
GhPOD17/Gh_A03G1812	chrA03:99201877-99205979	4103/867	288/31.94/7.2	N/N/-
GhPOD18/Gh_A03G2152	chrA03:16442-17606	1165/984	327/35.9/8.94	S/N/S
GhPOD19/Gh_A03G2153	chrA03:28781-29958	1178/987	328/35.15/7.68	S/N/S
GhPOD20/Gh_A04G0639	chrA04:44156365-44158523	2159/990	329/37.28/7.27	S/N/S
GhPOD21/Gh_A04G0963	chrA04:59248781-59249939	1159/996	331/36/8.26	S/N/S
GhPOD22/Gh_A04G1453	chrA04:7944-10075	2132/1020	339/37.62/7.73	N/S/S
GhPOD23/Gh_A05G0093	chrA05:1157788-1159020	1233/963	320/34.93/7.34	S/N/S
GhPOD24/Gh_A05G0507	chrA05:5454946-5456283	1338/999	332/36.14/5.05	N/N/S
GhPOD25/Gh_A05G0661	chrA05:6938388-6950337	11950/1014	337/36.17/6.73	S/N/S
GhPOD26/Gh_A05G0863	chrA05:8607073-8608956	1884/753	250/27.59/6.35	N/S/-
GhPOD27/Gh_A05G1328	chrA05:13602610-13604358	1749/1053	350/38.23/9.51	S/N/S
GhPOD28/Gh_A05G1452	chrA05:15020810-15022360	1551/999	332/37.66/8.54	S/N/S
GhPOD29/Gh_A05G1479	chrA05:15196748-15197863	1116/1011	336/36.82/8.25	N/S/-
GhPOD30/Gh_A05G1577	chrA05:16171654-16172991	1338/951	316/34.19/9.5	S/N/S
GhPOD31/Gh_A05G1635	chrA05:16892245-16893617	1373/1050	349/37.73/7.7	S/N/S
GhPOD32/Gh_A05G2401	chrA05:29807925-29809249	1325/972	323/34.96/9.47	S/N/S
GhPOD33/Gh_A05G2945	chrA05:72277688-72279245	1558/972	323/35.2/4.67	S/N/S
GhPOD34/Gh_A05G3141	chrA05:81059455-81060420	966/966	321/35.48/8.86	S/N/S
GhPOD35/Gh_A05G3239	chrA05:84740299-84741875	1577/972	323/35.83/5.06	N/S/S
GhPOD36/Gh_A05G3489	chrA05:90428029-90429659	1631/1014	337/36.06/9.57	S/N/S
GhPOD37/Gh_A05G3726	chrA05:122462-125928	3467/1182	393/42.73/9.35	N/S/C
GhPOD38/Gh_A06G0019	chrA06:84546-85807	1262/954	317/34.23/9.28	S/N/S
GhPOD39/Gh_A06G0383	chrA06:6376778-6380906	4129/1167	388/42.25/7.68	N/S/C
GhPOD40/Gh_A06G0929	chrA06:38044180-38045572	1393/1038	345/37.15/4.4	S/N/S
GhPOD41/Gh_A06G1006	chrA06:49554553-49556908	2356/987	328/35.73/8.27	S/N/S
GhPOD42/Gh_A06G2046	chrA06:45925-48981	3057/960	319/34.42/8.88	N/S/C
GhPOD43/Gh_A07G0012	chrA07:136360-137613	1254/996	331/36.45/9.58	S/N/S
GhPOD44/Gh_A07G0275	chrA07:3399593-3400898	1306/996	331/35.82/4.82	S/N/-
GhPOD45/Gh_A07G1997	chrA07:76170501-76172204	1704/1026	341/37.65/8.37	S/N/S
GhPOD46/Gh_A07G2090	chrA07:77469444-77471552	2109/1101	366/40.24/6.74	N/S/-
GhPOD47/Gh_A07G2109	chrA07:77657753-77658858	1106/966	321/34.93/4.26	S/N/S
GhPOD48/Gh_A07G2110	chrA07:77659654-77660804	1151/975	324/35.43/7.68	S/N/S
GhPOD49/Gh_A08G0345	chrA08:4189416-4190503	1088/1002	333/35.9/7.9	S/N/S
GhPOD50/Gh_A08G0347	chrA08:4289654-4290385	732/732	243/25.94/7.89	N/S/-
GhPOD51/Gh_A08G0711	chrA08:17779406-17781156	1751/999	332/35.79/5.71	N/S/S
GhPOD52/Gh_A08G0712	chrA08:18361761-18367521	5761/1056	351/38.2/4.88	S/N/S
GhPOD53/Gh_A08G0714	chrA08:18413356-18418531	5176/1032	343/36.85/4.51	S/N/S
GhPOD54/Gh_A08G0747	chrA08:24039157-24042529	3373/990	329/36.31/10.43	S/N/S
GhPOD55/Gh_A08G1744	chrA08:97409160-97411219	2060/753	250/27.54/5.6	N/N/-
GhPOD56/Gh_A08G1745	chrA08:97440700-97442620	1921/726	241/26.72/7.74	N/N/-
GhPOD57/Gh_A08G1746	chrA08:97442829-97446411	3583/867	288/32.07/6.91	N/N/-
GhPOD58/Gh_A08G1806	chrA08:98615798-98617217	1420/960	319/34.42/5.68	S/N/S
GhPOD59/Gh_A08G1950	chrA08:100557586-100558616	1031/951	316/34.48/9.77	S/N/S
GhPOD60/Gh_A08G2028	chrA08:101461027-101462441	1415/969	322/34.96/7.75	S/N/S
GhPOD61/Gh_A09G0591	chrA09:48054359-48058181	3823/777	258/28.28/8.82	N/S/-
GhPOD62/Gh_A09G1202	chrA09:63774095-63775448	1354/1020	339/36.01/5.24	S/N/S
GhPOD63/Gh_A09G1415	chrA09:67412511-67414343	1833/990	329/37.12/6.58	S/N/S
GhPOD64/Gh_A09G2334	chrA09:93316-94482	1167/951	316/34.58/10.02	S/N/S
GhPOD65/Gh_A09G2396	chrA09:35011-35983	973/906	301/33.44/8.46	S/N/S
GhPOD66/Gh_A10G0565	chrA10:7031511-7032708	1198/1041	346/38.29/6.03	S/N/S
GhPOD67/Gh_A10G0810	chrA10:16610264-16611518	1255/993	330/35.91/9.88	S/N/M
GhPOD68/Gh_A10G1317	chrA10:69770818-69772754	1937/966	321/33.76/8.33	S/N/S
GhPOD69/Gh_A10G1318	chrA10:69892772-69893920	1149/954	317/34.64/9.07	S/N/S
GhPOD70/Gh_A10G1537	chrA10:84415649-84417960	2312/972	323/35.59/8.64	S/N/S
GhPOD71/Gh_A10G1626	chrA10:87538433-87544412	5980/909	302/33.3/10.36	N/S/-
GhPOD72/Gh_A10G1627	chrA10:87544899-87546095	1197/987	328/35.3/9.64	S/N/S
GhPOD73/Gh_A10G2288	chrA10:1167442-1169647	2206/984	327/35.78/10.03	S/N/S
GhPOD74/Gh_A10G2290	chrA10:1189614-1190983	1370/993	330/36.03/8.94	S/N/S
GhPOD75/Gh_A11G0400	chrA11:3718938-3720282	1345/996	331/36.51/9.37	S/N/S
GhPOD76/Gh_A11G0523	chrA11:4917637-4918870	1234/927	308/34.89/9.73	N/S/S
GhPOD77/Gh_A11G1669	chrA11:25291980-25293232	1253/1005	334/36.93/8.46	S/N/S
GhPOD78/Gh_A11G1859	chrA11:45617267-45622682	5416/930	309/34.02/4.72	N/N/-
GhPOD79/Gh_A11G2043	chrA11:63260806-63261288	483/483	160/17.1/4.89	N/S/-
GhPOD80/Gh_A11G3132	chrA11:6967-8208	1242/966	321/34.97/8	S/N/S
GhPOD81/Gh_A12G0055	chrA12:735887-756659	20773/972	323/35.31/8.26	S/N/S
GhPOD82/Gh_A12G0056	chrA12:760206-761426	1221/972	323/35.56/8.99	S/N/S
GhPOD83/Gh_A12G0695	chrA12:26988350-26989090	741/561	186/20.48/6.66	N/S/-
GhPOD84/Gh_A12G0795	chrA12:45772636-45776292	3657/1068	355/39.81/5.12	S/N/S
GhPOD85/Gh_A12G1441	chrA12:73250331-73252001	1671/1023	340/37.17/9.36	N/N/M
GhPOD86/Gh_A12G1915	chrA12:81887117-81888429	1313/1014	337/37.91/6	S/N/S
GhPOD87/Gh_A12G2221	chrA12:84939580-84946625	7046/3291	1096/122.66/7.51	N/N/-
GhPOD88/Gh_A12G2370	chrA12:86207288-86208988	1701/972	323/35.63/8.88	S/N/S
GhPOD89/Gh_A12G2508	chrA12:87451770-87452774	1005/1005	334/37.17/7.73	S/N/S
GhPOD90/Gh_A12G2622	chrA12:49766-51061	1296/1011	336/37.08/5.05	S/N/S
GhPOD91/Gh_A13G0039	chrA13:393916-396005	2090/978	325/35.42/9.01	S/N/S
GhPOD92/Gh_A13G0772	chrA13:33291935-33293456	1522/975	324/34.9/6.65	S/N/S
GhPOD93/Gh_A13G0773	chrA13:33310901-33312163	1263/987	328/35.42/7.36	S/N/S
GhPOD94/Gh_A13G2003	chrA13:79484281-79487307	3027/762	253/28/5.19	N/N/-
GhPOD95/Gh_D01G0112	chrD01:855518-857368	1851/975	324/35.25/9.56	S/N/-
GhPOD96/Gh_D01G1310	chrD01:36863811-36864806	996/996	331/36.9/8.19	S/N/S
GhPOD97/Gh_D01G1317	chrD01:37193921-37194913	993/993	330/36.56/7.96	S/N/S
GhPOD98/Gh_D01G1359	chrD01:40012715-40014005	1291/969	322/34.7/7.93	S/N/S
GhPOD99/Gh_D01G1632	chrD01:51431707-51437024	5318/867	288/31.78/5.81	N/S/-
GhPOD100/Gh_D01G1726	chrD01:53949584-53951128	1545/972	323/34.9/5.4	S/N/S
GhPOD101/Gh_D02G0606	chrD02:8255229-8256474	1246/1014	337/36.66/4.13	S/N/S
GhPOD102/Gh_D02G0695	chrD02:9863175-9865761	2587/1116	371/40.62/7.7	N/S/M
GhPOD103/Gh_D02G1110	chrD02:31109209-31111160	1952/1020	339/37.91/8.45	S/N/S
GhPOD104/Gh_D02G1327	chrD02:43855329-43856508	1180/1008	335/36.76/5.38	S/N/S
GhPOD105/Gh_D02G1345	chrD02:44875923-44877373	1451/990	329/36.19/9.18	S/N/S
GhPOD106/Gh_D02G1346	chrD02:44991381-44992620	1240/984	327/35.44/7.72	S/N/S
GhPOD107/Gh_D02G1997	chrD02:64059836-64065386	5551/987	328/35.24/7.94	S/N/S
GhPOD108/Gh_D02G1998	chrD02:64081876-64083040	1165/984	327/35.7/9.37	S/N/S
GhPOD109/Gh_D02G2245	chrD02:66344682-66348942	4261/867	288/31.96/7.2	N/N/-
GhPOD110/Gh_D03G0059	chrD03:390493-391719	1227/954	317/34.27/9.27	S/N/S
GhPOD111/Gh_D03G0074	chrD03:531888-534705	2818/912	303/33.96/8.22	N/S/-
GhPOD112/Gh_D03G0246	chrD03:2618435-2620271	1837/993	330/35.99/9.44	S/N/S
GhPOD113/Gh_D03G0641	chrD03:19207034-19208004	971/699	232/25.15/8.87	N/N/S
GhPOD114/Gh_D03G1381	chrD03:42417094-42420536	3443/1158	385/42.12/7.65	N/S/-
GhPOD115/Gh_D03G1382	chrD03:42426148-42428382	2235/969	322/34.55/9.72	S/N/S
GhPOD116/Gh_D04G0130	chrD04:1796590-1798244	1655/1014	337/36.04/9.22	S/N/S
GhPOD117/Gh_D04G0735	chrD04:15156949-15158096	1148/972	323/35.41/8.64	S/N/S
GhPOD118/Gh_D04G1101	chrD04:36441991-36444139	2149/987	328/37.19/6.73	S/N/S
GhPOD119/Gh_D04G1116	chrD04:36784560-36786018	1459/723	240/26.55/6.51	N/N/-
GhPOD120/Gh_D04G1506	chrD04:46932159-46969978	37820/2016	671/72.76/7.71	S/N/S
GhPOD121/Gh_D04G1593	chrD04:48023771-48025856	2086/1020	339/37.4/7.06	N/N/S
GhPOD122/Gh_D05G0154	chrD05:1545607-1546840	1234/978	325/35.03/7.99	S/N/S
GhPOD123/Gh_D05G0626	chrD05:5026931-5028271	1341/1002	333/36.15/5.05	N/N/S
GhPOD124/Gh_D05G0807	chrD05:6771535-6772831	1297/1014	337/36.1/6.73	S/N/S
GhPOD125/Gh_D05G1498	chrD05:13463061-13464814	1754/1059	352/38.37/9.62	N/S/C
GhPOD126/Gh_D05G1626	chrD05:14640100-14641647	1548/999	332/37.65/8.54	S/N/S
GhPOD127/Gh_D05G1755	chrD05:15837493-15838825	1333/951	316/34.22/9.47	S/N/S
GhPOD128/Gh_D05G1817	chrD05:16541131-16542504	1374/1050	349/37.76/7.7	S/N/S
GhPOD129/Gh_D05G2244	chrD05:21543390-21547747	4358/1218	405/43.82/9.15	N/S/C
GhPOD130/Gh_D05G2666	chrD05:27862573-27863898	1326/972	323/34.85/8.92	S/N/S
GhPOD131/Gh_D05G3256	chrD05:51004089-51005627	1539/951	316/33.69/8.5	S/N/S
GhPOD132/Gh_D05G3875	chrD05:76982-78848	1867/753	250/27.57/6.04	N/S/-
GhPOD133/Gh_D06G0413	chrD06:5889650-5894261	4612/1353	450/49.69/7.3	N/S/C
GhPOD134/Gh_D06G1049	chrD06:22550424-22553640	3217/1008	335/36.1/9.95	N/S/C
GhPOD135/Gh_D06G1170	chrD06:28537753-28539152	1400/1038	345/37.22/4.46	S/N/S
GhPOD136/Gh_D06G1200	chrD06:30337737-30340827	3091/1050	349/37.92/8.39	S/N/S
GhPOD137/Gh_D07G0019	chrD07:200078-201330	1253/996	331/36.42/9.11	S/N/S
GhPOD138/Gh_D07G0331	chrD07:3515650-3516955	1306/996	331/35.86/4.66	S/N/S
GhPOD139/Gh_D07G1629	chrD07:32319582-32320538	957/792	263/28.65/9.4	N/S/-
GhPOD140/Gh_D07G1630	chrD07:32393320-32394431	1112/939	312/34.24/9.89	S/N/S
GhPOD141/Gh_D07G2217	chrD07:53125200-53126909	1710/1026	341/37.7/8.37	S/N/S
GhPOD142/Gh_D07G2320	chrD07:54647068-54648167	1100/966	321/35.08/4.35	S/N/S
GhPOD143/Gh_D07G2321	chrD07:54648867-54650018	1152/975	324/35.39/7.24	S/N/S
GhPOD144/Gh_D08G0441	chrD08:4640562-4641585	1024/858	285/30.9/8.72	N/S/C
GhPOD145/Gh_D08G0442	chrD08:4670537-4671627	1091/1005	334/35.7/8.55	S/N/S
GhPOD146/Gh_D08G0443	chrD08:4687890-4688979	1090/1005	334/35.66/8.55	S/N/S
GhPOD147/Gh_D08G0829	chrD08:13789103-13790820	1718/999	332/35.8/6.5	S/N/S
GhPOD148/Gh_D08G0832	chrD08:14037040-14038366	1327/1065	354/38.22/4.6	S/N/S
GhPOD149/Gh_D08G2093	chrD08:59783046-59784995	1950/741	246/27.13/6.04	N/S/-
GhPOD150/Gh_D08G2094	chrD08:59804611-59806683	2073/738	245/27.03/5.9	N/N/-
GhPOD151/Gh_D08G2095	chrD08:59806893-59810058	3166/879	292/32.56/6.95	N/N/-
GhPOD152/Gh_D08G2167	chrD08:61090294-61091717	1424/960	319/34.38/5.68	S/N/S
GhPOD153/Gh_D08G2330	chrD08:63032857-63033881	1025/945	314/34.22/9.44	S/N/S
GhPOD154/Gh_D08G2420	chrD08:64145436-64146846	1411/969	322/34.92/7.75	S/N/S
GhPOD155/Gh_D09G0590	chrD09:28473937-28477562	3626/990	329/36.6/9.74	S/N/S
GhPOD156/Gh_D09G1208	chrD09:39009612-39010932	1321/1020	339/35.98/4.95	S/N/S
GhPOD157/Gh_D09G1420	chrD09:41767263-41769101	1839/987	328/36.92/6.28	S/N/S
GhPOD158/Gh_D09G1611	chrD09:43695508-43696494	987/987	328/36.13/6.76	S/N/S
GhPOD159/Gh_D09G2046	chrD09:47806422-47807579	1158/951	316/34.5/9.58	S/N/S
GhPOD160/Gh_D09G2047	chrD09:47809072-47810196	1125/885	294/31.91/9.35	S/N/S
GhPOD161/Gh_D09G2048	chrD09:47810407-47821212	10806/2019	672/73.64/9.25	S/N/S
GhPOD162/Gh_D09G2418	chrD09:1692-2815	1124/1026	341/37.69/9.36	S/N/S
GhPOD163/Gh_D10G0605	chrD10:6317691-6318891	1201/1041	346/38.32/6.12	S/N/S
GhPOD164/Gh_D10G0951	chrD10:12771913-12773186	1274/993	330/36.01/9.99	S/N/M
GhPOD165/Gh_D10G1157	chrD10:19241093-19242236	1144/954	317/34.55/9.06	S/N/S
GhPOD166/Gh_D10G1158	chrD10:19299752-19300772	1021/705	234/24.46/8.21	N/S/-
GhPOD167/Gh_D10G1643	chrD10:45423530-45424891	1362/993	330/36.1/8.98	S/N/S
GhPOD168/Gh_D10G1644	chrD10:45445937-45448111	2175/984	327/35.72/10.04	S/N/S
GhPOD169/Gh_D10G1784	chrD10:50389073-50391342	2270/972	323/35.64/8.8	S/N/S
GhPOD170/Gh_D10G1880	chrD10:52703553-52704762	1210/1041	346/37.99/8.78	S/N/S
GhPOD171/Gh_D10G1881	chrD10:52813459-52814911	1453/972	323/35.59/10.39	S/N/S
GhPOD172/Gh_D10G1882	chrD10:52815399-52816603	1205/987	328/35.25/9.64	S/N/S
GhPOD173/Gh_D11G0463	chrD11:3963558-3964899	1342/996	331/36.57/9.22	S/N/S
GhPOD174/Gh_D11G0607	chrD11:5302020-5303236	1217/978	325/36.13/8.95	N/S/S
GhPOD175/Gh_D11G1824	chrD11:20830683-20831933	1251/969	322/35.55/8.46	S/N/S
GhPOD176/Gh_D11G2151	chrD11:32727567-32730117	2551/972	323/35.51/4.66	S/N/S
GhPOD177/Gh_D11G2183	chrD11:34071064-34072304	1241/966	321/34.9/7.74	S/N/S
GhPOD178/Gh_D11G2348	chrD11:45744028-45745072	1045/957	318/35.11/10.5	S/N/M
GhPOD179/Gh_D12G0069	chrD12:815867-853649	37783/1764	587/64.17/7.56	N/S/-
GhPOD180/Gh_D12G0070	chrD12:860573-861841	1269/972	323/35.07/6.5	S/N/S
GhPOD181/Gh_D12G0071	chrD12:866590-867867	1278/1029	342/37.73/8.99	N/S/S
GhPOD182/Gh_D12G0072	chrD12:876401-877438	1038/789	262/28.85/8.06	N/S/-
GhPOD183/Gh_D12G0699	chrD12:15282699-15283971	1273/993	330/35.78/8.49	S/N/S
GhPOD184/Gh_D12G0788	chrD12:22644947-22646101	1155/1068	355/39.65/4.9	S/N/S
GhPOD185/Gh_D12G0853	chrD12:27599646-27601009	1364/1050	349/38.71/6.76	S/N/S
GhPOD186/Gh_D12G1559	chrD12:46570546-46572226	1681/1023	340/37.12/9.36	N/S/M
GhPOD187/Gh_D12G1577	chrD12:46856738-46864694	7957/1803	600/68.11/6.77	N/S/-
GhPOD188/Gh_D12G2095	chrD12:53969820-53971114	1295/1002	333/37.49/5.48	S/N/S
GhPOD189/Gh_D12G2504	chrD12:57927732-57929447	1716/972	323/35.48/8.72	S/N/S
GhPOD190/Gh_D12G2635	chrD12:59085174-59086178	1005/1005	334/37.2/7.99	S/N/S
GhPOD191/Gh_D13G0054	chrD13:451962-454035	2074/978	325/35.63/9.66	S/N/S
GhPOD192/Gh_D13G0906	chrD13:18641774-18643302	1529/978	325/34.95/6.77	S/N/S
GhPOD193/Gh_D13G0907	chrD13:18659873-18661135	1263/987	328/35.47/7.41	S/N/S
GhPOD194/Gh_D13G0909	chrD13:18677172-18678434	1263/987	328/35.5/7.41	S/N/S
GhPOD195/Gh_D13G2402	chrD13:60076810-60079762	2953/750	249/27.31/5.17	N/N/-
GhPOD196/Gh_Sca005203G02	chrSca:12082-12672	591/591	196/22.12/8.79	N/S/-
GhPOD197/Gh_Sca005268G01	chrSca:3248-4662	1415/960	319/34.49/7.93	S/N/S
GhPOD198/Gh_Sca007173G01	chrSca:8026-8850	825/825	275/30.21/6.74	S/N/S

Chr: chromosome; CDS: coding sequence; PL: peptide length; MW: molecular weight; N: non-secreted into the apoplast; S: secreted into apoplast with signal peptide in SignalP, without signal peptide but with non-classical secretion mode in SeretomeP and with signal peptide in TargetP; C: chloroplast; M: mitochondria; -: unsure location).

**Table 2:** The Ka, Ks, and Ka/Ks values for the 27 gene pairs.

Paralogous Pairs	Ks	Ka	Ka/Ks	Duplicate Type
Gh_A01G1388-Gh_A03G1812	0.446	0.065	0.15	Segmental
Gh_D03G0246-Gh_D12G0699	1.037	0.099	0.10	Segmental
Gh_D04G0130-Gh_D05G2666	0.573	0.086	0.15	Segmental
Gh_D04G1101-Gh_D09G1420	0.452	0.095	0.21	Segmental
Gh_D05G3875-Gh_D08G2093	0.373	0.03	0.08	Segmental
Gh_D07G1630-Gh_D09G2046	0.298	0.063	0.21	Segmental
Gh_D07G1630-Gh_D09G2418	0.205	0.046	0.22	Segmental
Gh_D08G2420-Gh_D11G2183	0.479	0.066	0.14	Segmental
Gh_A04G0639-Gh_A09G1415	0.486	0.095	0.20	Segmental
Gh_A05G0863-Gh_A08G1744	0.148	0.074	0.50	Segmental
Gh_A05G1577-Gh_A06G0019	0.508	0.07	0.14	Segmental
Gh_A05G2945-Gh_A07G2109	0.79	0.096	0.12	Segmental
Gh_A05G3141-Gh_A07G1997	0.721	0.079	0.11	Segmental
Gh_A08G2028-Gh_A11G3132	0.483	0.073	0.15	Segmental
Gh_D01G1632-Gh_D02G2245	0.452	0.063	0.14	Segmental
Gh_D08G2093-Gh_D08G2094	0.087	0.011	0.13	Tandem
Gh_D08G2094-Gh_D08G2095	0.087	0.017	0.20	Tandem
Gh_D09G2046-Gh_D09G2047	0.355	0.046	0.13	Tandem
Gh_D11G0463-Gh_D11G0607	0.129	0.039	0.30	Tandem
Gh_D13G0906-Gh_D13G0907	0.205	0.07	0.34	Tandem
Gh_D13G0907-Gh_D13G0909	0.009	0.001	0.11	Tandem
Gh_A08G1744-Gh_A08G1745	0.039	0.039	1.00	Tandem
Gh_A08G1745-Gh_A08G1746	0.033	0.033	1.00	Tandem
Gh_A10G2288-Gh_A10G2290	0.306	0.093	0.30	Tandem
Gh_A12G0055-Gh_A12G0056	0.289	0.061	0.21	Tandem
Gh_A13G0772-Gh_A13G0773	0.205	0.076	0.37	Tandem
Gh_D01G1310-Gh_D01G1317	0.018	0.008	0.44	Tandem
